# Chinese Students’ Perceptions of the Motivational Climate in College English Courses: Relationships Between Course Perceptions, Engagement, and Achievement

**DOI:** 10.3389/fpsyg.2022.853221

**Published:** 2022-05-23

**Authors:** Ming Li, Brett D. Jones, Thomas O. Williams, Yingjian Guo

**Affiliations:** ^1^School of Foreign Languages, Shanghai University of Engineering Sciences, Shanghai, China; ^2^School of Education, Virginia Tech, Blacksburg, VA, United States; ^3^School of Foreign Languages, Renmin University of China, Beijing, China

**Keywords:** motivation, engagement, MUSIC Model of Motivation, English courses, course perceptions, motivational climate, foreign language instruction, English as a second language

## Abstract

Effective teachers create a motivational climate that engages students in course activities in ways that lead to increased learning and achievement. Although researchers have identified motivational climate variables that are associated with students’ engagement and achievement, less is known about how these variables are related in different courses and cultures. The purpose of the two studies presented in this paper was to contribute to this research literature by examining these associations within the context of college English courses in two Chinese universities. Specifically, we investigated the relationships between students’ perceptions of the motivational climate (i.e., perceptions of empowerment/autonomy, usefulness, success, interest, and caring), cognitive and behavioral engagement, and achievement. This is the first study to examine the connections between all of these variables in one path model in college English courses in China. We administered surveys at two different Chinese universities (*n* = 332 and 259) and used regression and path analysis to examine the relationships among the variables. We demonstrated that (a) students’ perceptions of the motivational climate were related to their cognitive engagement, (b) cognitive engagement was related to their behavioral engagement, and (c) behavioral engagement predicted their achievement. These findings are consistent with and extend the growing body of literature on motivational climate and engagement, and they highlight the importance of some motivational climate perceptions over others as significant predictors of cognitive engagement. We conclude that effective English language teachers in China do the following: help students to believe that they can be successful, trigger and maintain students’ interest, and empower students by providing them with choices in activities and assignments.

## Introduction

Effective teachers engage students in course activities (Christenson et al., 2012b), which is important because students’ engagement is related to their achievement (Muenks et al., 2017; Tao et al., 2022). Researchers have identified a variety of factors associated with student engagement in courses, including their perceptions of the motivational climate (see Christenson et al., 2012b; Jones et al., 2021). Yet, the role of engagement as a link between students’ motivational climate perceptions and their achievement remains unclear, in part, because researchers do not always agree on the exact definitions of engagement or the order in which different types of engagement occur (Reschly and Christenson, 2012). For example, it has been suggested that cognitive engagement precedes behavioral engagement (Reschly and Christenson, 2012; Reeve et al., 2020), and this suggestion has been documented empirically by researchers (Jones and Carter, 2019).

The purpose of the present studies was to examine associations between university students’ motivational climate perceptions, engagement, and achievement in English language (EL) courses in China. We chose this context for a couple reasons. All Chinese college students are required to enroll in college EL courses and complete national English tests (Guo et al., 2020). Thus, understanding how students’ perceptions of the motivational climate in EL courses are related to their engagement in these courses—and subsequently achievement in these courses—could be useful to the large number of instructors who teach these courses. In addition, despite the importance of college EL courses, many Chinese students are unmotivated in these courses (Li et al., 2016; Li, 2021) and lack the skills needed to pass the national exams (Hertling, 1996). Understanding how students’ motivation-related perceptions in EL courses affect their engagement and achievement could lead to the development of effective instructional strategies and interventions aimed at engaging students.

More specifically, the present studies can contribute to the literature about effective teaching in two ways. First, the results will determine whether the motivational climate constructs that have been shown to affect students’ engagement in other contexts also affect students’ engagement in EL courses in China. Relatedly, the results will identify which motivational climate constructs are most salient in this context. Second, the results will determine whether the motivational climate constructs and engagement can be linked to achievement in this context; and if so, whether cognitive engagement precedes behavioral engagement as hypothesized. Together, these findings will provide a clearer understanding of the motivational climate factors that can affect students’ engagement in EL courses in China. Teachers can then focus on incorporating teaching strategies related to these factors in order to increase students’ engagement and achievement.

## Literature Review

### Motivation and Engagement in Courses

Engagement is a broad psychological construct that has multiple definitions and has been studied in a variety of contexts. Many researchers consider motivation to precede engagement and define motivation as one’s intentions to engage (Jones, 2018, 2020b) and engagement as one’s actions (Christenson et al., 2012a). Engagement can be further divided into a few dimensions, including behavioral engagement (e.g., effort, actual participation in school and learning), cognitive engagement (e.g., cognitive investment in the coursework, such as mental effort and use of effective learning strategies), and emotional engagement (e.g., students’ emotional responses to teachers, peers, and the school environment, such as enjoyment and anxiety; Fredricks et al., 2004). Students’ engagement predicts many different positive educational outcomes, such as achievement, learning, and the likelihood of high school completion (Reschly and Christenson, 2012; Skinner and Pitzer, 2012; Tao et al., 2022).

Many different psychological theories have been used to explain students’ engagement in educational settings, such as self-determination theory (Reeve, 2012; Ryan and Deci, 2020), social cognitive theory (Bandura, 1997; Schunk and DiBenedetto, 2020), self-regulation theories (Cleary and Zimmerman, 2012), theories of emotions (Pekrun and Linnenbrink-Garcia, 2012), and interest theories (Ainley, 2012; Renninger and Hidi, 2015; for more perspectives, see Christenson et al., 2012b). Simultaneously, a mostly separate research literature has developed to explain the motivation of students in second/foreign language (L2) courses (Al-Hoorie, 2017; Al-Hoorie and MacIntyre, 2019). This research has led to notable contributions such as the Socio-educational Model of Second Language Acquisition by Gardner (2019) and the L2 motivational self-system by Dörnyei (2009). Although L2 researchers have made some connections between teachers’ motivational strategies, students’ motivation, students’ engagement, and students’ achievement (e.g., Alrabai, 2016), the research directly related to students’ perceptions in a course and the effects of these perceptions on students’ engagement and achievement has been limited (see Lamb, 2019, for a review). Instead, L2 researchers have focused on studying the motivation of students, as opposed to focusing on motivating students in courses (Boo et al., 2015; Lamb, 2019).

Recently, Jones (2020a) has suggested that the MUSIC Model of Motivation (abbreviated in this paper as the MUSIC model; Jones, 2009, 2018) could be applied to L2 instruction in a manner that “does not replace existing L2 motivation theories, but rather…used as a complementary approach” (p. 2). The multidimensional MUSIC model highlights five student perceptions of the motivational climate—perceptions that can be linked to current motivation-related constructs and psychological theories—that have been shown to be associated with student engagement (Jones, 2010, 2019; Jones et al., 2021) and course ratings (Wilkins et al., 2021; Jones et al., 2022b). The five motivational climate perceptions include students’ perceptions of: their autonomy/empowerment in the class (*eMpowerment*), the usefulness/utility value of the content and activities (*Usefulness*), the extent to which they can be successful if they put forth effort (*Success*), their enjoyment/interest during the activity (*Interest*), and whether the instructor and other students care about their learning and about them personally (*Caring*; the five keywords form the acronym MUSIC: eMpowerment, Usefulness, Success, Interest, and Caring). Evidence that these perceptions affect students’ motivation and engagement is provided by a variety of theories, including arousal theories (Berlyne, 1960), attachment theory (Bowlby, 1969), attribution theory (Weiner, 2000), situated expectancy-value theory (Eccles and Wigfield, 2020), interest theories (Schraw and Lehman, 2001; Renninger and Hidi, 2015), self-determination theory (Ryan and Deci, 2020), social cognitive theory (Bandura, 1997; Schunk and DiBenedetto, 2020), among others (see Jones, 2018 for a more comprehensive list). Examples of empirical research studies linking these five motivational climate perceptions and student engagement include the following: Giving students’ autonomy (empowerment) has been shown to increase student engagement (Reeve et al., 2004; Jang et al., 2012), perceived instrumentality (usefulness) and self-efficacy (success) were found to increase engagement (Walker and Greene, 2009), curiosity (situational interest) has been associated with students engagement in science (Wu and Wu, 2020), and several studies have shown that the caring relationship between a teacher and students leads to higher student engagement and achievement (King, 2015; Quin, 2016). Thus, the MUSIC model constructs are consistent with current psychological constructs and theories. In addition, the MUSIC constructs are consistent with motivational teaching strategies proposed by L2 researchers (e.g., Dörnyei and Ushioda, 2011), such as the need for instructors to: support learner autonomy (empowerment), help students to connect the relevance of course activities to their lives (usefulness), increase learners’ expectancy of success (success), get students interested in course activities, and foster relationships between teachers and students (caring).

### MUSIC Perceptions and Engagement

**Figure 1:**
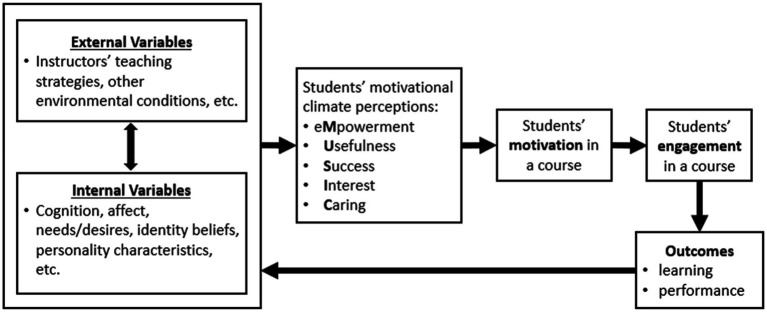
Key elements of the MUSIC model of motivation. Adapted from “Motivating Students by Design: Practical Strategies for Professors” by Jones (2018). Used with permission.

Figure 1 shows how, in the MUSIC model, external and internal variables affect students’ perceptions of the motivational climate in a course, which then affects their motivation, engagement, and learning/performance. Although students have a variety of perceptions within a course, their MUSIC perceptions (i.e., perceptions of empowerment, usefulness, success, interest, and caring) have been studied because they relate to important outcomes and have been shown to be distinct; that is, they refer to different constructs that are separable through factor analyses (Mohamed et al., 2013; Jones et al., 2014, 2016, 2019; Jones and Sigmon, 2016; Pace et al., 2016; Schram and Jones, 2016; Chittum and Jones, 2017; Tendhar et al., 2017; Gladman et al., 2020).

The MUSIC perceptions have been shown to be correlated to their students’ engagement in studies of undergraduate students (Jones, 2010, 2019; Jones et al., 2021), and several decades of research have documented that constructs related to these perceptions are related to students’ engagement (for reviews, see Christenson et al., 2012a; Schunk et al., 2014). Yet, studies that have included all five MUSIC perceptions sometimes find that some, but not all, of the MUSIC perceptions are related to student engagement in different educational contexts. For example, in a study of college courses, Jones (2019) documented that all five MUSIC perceptions were related to students’ behavioral engagement in some courses, but that only two, three, or four of the MUSIC perceptions were related to behavioral engagement in other courses. Within EL courses in China, only one study (Li et al., 2016) has examined the relationship between all five MUSIC perceptions and students’ engagement. This study showed that although empowerment, usefulness, success, and interest (but not caring) were correlated with student engagement, only empowerment and success were significantly related to engagement when all of these variables were included in one regression model to predict engagement. This study was limited to 101 students in three classes at one college; and therefore, more studies replicating this study in other contexts would be useful.

In addition, only two studies (Jones and Carter, 2019; Jones et al., 2021) have included constructs related to all five MUSIC perceptions, engagement, and learning/achievement. The Jones and Carter study was conducted within a psychology course at a university in the United States and demonstrated that while all five MUSIC constructs were significantly correlated with students’ cognitive and behavioral engagement, only empowerment and usefulness were significantly related to cognitive engagement when all the constructs were included in one model. Furthermore, cognitive and behavioral engagement were significantly related to student learning; and importantly, the MUSIC constructs predicted cognitive engagement, which was modeled to precede behavioral engagement and then learning. In Jones et al. (2021) study, the MUSIC perceptions of students in an online geography course at a United States university were positively correlated with their effort in the course. When all five MUSIC constructs were included in a structural equation model with MUSIC perceptions predicting behavioral engagement, only interest and caring were significant predictors of behavioral engagement, which then predicted achievement. Findings from studies such as these demonstrate that different course perceptions can be more influential in some courses than others. Relationships between these variables need to be better understood because if patterns are found in EL courses in China (e.g., usefulness is most strongly related to engagement and achievement), they could be used to help instructors to design courses that target these perceptions to lead to increased student engagement and achievement. Therefore, the purpose of the present studies was to identify the MUSIC perceptions that are most highly associated with students’ engagement in EL courses in China.

## Purpose and Research Questions

More research is needed within specific contexts (e.g., different types of courses at different colleges) to document which MUSIC perceptions are most salient in different contexts. For example, empowerment and usefulness may be most important to engaging students in a psychology course in the United States (as demonstrated in the study by Jones and Carter, 2019), but perceptions of empowerment and success might be more important in a college English language course in China (as demonstrated in the study by Li et al., 2016). The aim of the present studies was to investigate the effects of students’ MUSIC perceptions in the context of college EL courses in China to better understand (a) which MUSIC perceptions are most important in predicting cognitive and behavioral engagement in EL courses in China, (b) the extent to which cognitive and behavioral engagement in EL courses are related to EL achievement, and (c) whether cognitive engagement precedes behavioral engagement as mediators between MUSIC perceptions and achievement. The results of these studies could help EL instructors to become more effective by identifying the components of the motivational climate that are most important in engaging students in their courses. Teachers could then design instructional activities to support these components of the motivational climate.

In the present studies, we chose to focus on cognitive and behavioral engagement instead of emotional engagement for a few reasons. First, emotional engagement overlaps conceptually with situational interest, which is one of the course perceptions already included in the MUSIC model. In the MUSIC model, situational interest is defined similar to how other researchers have defined it, as “liking and willful engagement in a cognitive activity” (Schraw and Lehman, 2001, p. 23). This definition includes an affective component (the liking) similar to emotional engagement—which includes students’ emotional responses such as enjoyment (Fredricks et al., 2004)—and includes a willful engagement. Reeve et al. (2020) provided empirical evidence of the conceptual overlap between emotional engagement and interest/intrinsic motivation. Second, emotional engagement likely precedes engagement or “amplifies on-going and future behavioral engagement” (Reeve et al., 2020, p. 8). And finally, emotional engagement does not consistently predict educational outcomes such as achievement (Gutiérrez and Tomás, 2019; Reeve et al., 2020).

We conducted two studies to examine the extent to which students’ MUSIC perceptions in an English language course in China affect their cognitive engagement, behavioral engagement, and achievement. Our five specific research questions were as follows:

- RQ1 (Study 1 and Study 2): To what extent do students’ MUSIC perceptions relate to their cognitive engagement?
- RQ2 (Study 1 and Study 2): To what extent do students’ MUSIC perceptions relate to their behavioral engagement?
- RQ3 (Study 2): To what extent do students’ MUSIC perceptions positively predict their cognitive engagement, which then positively predicts their achievement?
- RQ4 (Study 2): To what extent do students’ MUSIC perceptions positively predict their behavioral engagement, which then positively predicts their achievement?
- RQ5 (Study 2): To what extent do students’ MUSIC perceptions positively predict their cognitive engagement, which then positively predicts their behavioral engagement, which then positively predicts their achievement?

We conducted Study 1 to examine RQ1 and RQ2 as a proof of concept that one or more MUSIC perceptions were related to students’ cognitive and behavioral engagement in college English courses in China. After providing evidence of these relationships, we conducted Study 2 with a different sample of students to provide evidence that the results of Study 1 were generalizable to students attending another university and to answer RQ3, RQ4, and RQ5 by modeling the relationships between MUSIC perceptions, cognitive and behavioral engagement, and achievement.

We predicted that students’ MUSIC perceptions would be positively related to their cognitive and behavioral engagement based on studies that have documented these relationships with non-EL courses in the United States (e.g., Jones, 2019; Jones and Carter, 2019; Jones et al., 2021), students with non-EL courses in China (Jones et al., 2017), and studies with EL courses in China (Li et al., 2016). We also predicted that students’ cognitive and behavioral engagement would positively relate to their achievement based on studies that have shown these relationships with non-EL courses in the United States (e.g., Muenks et al., 2017; Di Leo et al., 2019; Jones and Carter, 2019). Our third prediction was that cognitive and behavioral engagement would mediate the relationship between students’ MUSIC perceptions and their achievement. We based this prediction on the reasoned hypotheses by some researchers (Reschly and Christenson, 2012; Reeve et al., 2020) and the empirical findings of others (Jones and Carter, 2019).

## Study 1

### Method

#### Participants

Participants in Study 1 were 332 undergraduate students at a large university in northeastern China. The students were enrolled in one of four English classes (*n* = 46, 76, 100, 110) for non-English majors. More of the participants were female (*n* = 250; 75.3%) than male (*n* = 82; 24.7%). The majority of the participants reported that they were Han nationality (*n* = 203, 61.1%), whereas 127 participants (38.3%) reported that they were one of the minority nationalities, and two students (0.6%) reported that they were an “other” race/ethnicity. Regarding their class standing, 77 (23.2%) were first-year students, 254 (76.5%) were sophomores, and 1 (0.3%) was a senior. Most students (*n* = 327; 98.5%) were between the ages of 18 and 22, and five students (1.5%) were older than 22 years old. Most or all students had been enrolled in English classes in school for at least 10 years (since elementary school).

#### Procedure

Students completed an online questionnaire near the end of the semester in their English course. Because all the participants were learning English (and thus, not fluent in English), all of the questionnaire items were provided in Chinese. Students completed the survey as part of their normal class activities; and therefore, consent to participate in this study was not obtained. Instead, we received the anonymous data from the instructors as existing data and their inclusion in this study was approved as “Exempt” by the authors’ Institutional Review Board (IRB #17-021).

#### Motivational Climate

We measured the motivational climate using the MUSIC® Model of Academic Motivation Inventory (College Student short-form version; available at Jones, 2012/2021), which consists of 20 items that form five scales: a four-item empowerment scale (measuring autonomy; Ryan and Deci, 2020), a four-item usefulness scale (measuring utility value; Eccles and Wigfield, 2020), a four-item success scale (measuring expectancy for success; Eccles and Wigfield, 2020), a four item interest scale (measuring situational interest; Renninger and Hidi, 2015), and a four-item caring scale (measuring caring; Noddings, 1992). All items were responded to on a six-point Likert-format scale: 1 = *Strongly disagree*, 2 = *Disagree*, 3 = *Somewhat disagree*, 4 = *Somewhat agree*, 5 = *Agree*, 6 = *Strongly agree*. Example items include: “I had flexibility in what I was allowed to do in this course” (empowerment), “In general, the coursework was useful to me” (usefulness), “I was confident that I could succeed in the coursework” (success), “The coursework was interesting to me” (interest), and “The instructor cared about how well I did in this course” (caring). The MUSIC Inventory produces reliable and valid scores and correlates with other measures as expected (Jones and Skaggs, 2016; Pace et al., 2016; Jones et al., 2019). The Chinese translation of the MUSIC Inventory has also been shown to demonstrate acceptable psychometric properties; for example, Cronbach’s alpha values for the scales were 0.82 for empowerment, 0.89 for usefulness, 0.87 for success, 0.93 for interest, and 0.88 for caring (Jones et al., 2017).

#### Behavioral Engagement

To measure behavioral engagement, we used a three-item effort scale that was based on the Effort/Importance scale, which is part of the Intrinsic Motivation Inventory (Ryan, 1982; McAuley et al., 1989). All items included a 6-point Likert-format scale (1 = *Strongly disagree*, 2 = *Disagree*, 3 = *Somewhat disagree*, 4 = *Somewhat agree*, 5 = *Agree*, 6 = *Strongly agree*). An example item is: “I put a lot of effort into this course.” In Jones (2010), the reliability estimates were good (*α* = 0.84, 0.84, 0.86, 0.84). We used the Chinese translation of this scale that was used in Jones et al. (2017).

#### Cognitive Engagement

We used the 8-item Self-Regulated Strategy Use scale that is part of the Student Perceptions of Classroom Knowledge-Building Scale (SPOCK; Shell et al., 2005; Shell and Husman, 2008) to assess cognitive engagement. The Self-Regulated Strategy Use scale measures the extent to which students’ behaviors and strategies are associated with self-regulation (e.g., planning, goal setting, monitoring, and evaluation of studying and learning). An example item is: “I try to determine the best approach for studying each assignment” (1 = *Almost never*, 2 = *Seldom*, 3 = *Sometimes*, 4 = *Often*, 5 = *Almost always,* 6 = *Always*). Reliability estimates have been shown to be acceptable (α = 0.81; Shell and Husman, 2008). We used the Chinese translation of this scale that was used in Jones et al. (2017).

#### Analysis

We used IBM® SPSS® version 26 to compute measures of dispersion, distribution, and correlation, and to conduct an exploratory factor analysis (EFA) to examine the psychometric properties of the MUSIC Inventory items. We used Amos version 25 to run regression analyses with the MUSIC constructs predicting cognitive engagement in one model and behavioral engagement in another. For all statistical tests, we set the alpha value at 0.05, and we report two-tailed values of *p*.

### Results

We conducted an EFA on the 20-item MUSIC Inventory using a principal factors analysis and a Promax rotation with Kaiser normalization (*n* = 332). We removed two items (an empowerment item and a usefulness item) because they loaded higher on a factor other than their intended factor. All the other items loaded on their factors as anticipated. Only three items cross-loaded on another factor at value greater than 0.20. We retained these three items because (a) the cross-loading values (i.e., 0.24, 0.32, and 0.34) were not very high (Tabachnick and Fidell, 2007), (b) the items have been shown to load on these factors in other studies (e.g., Jones et al., 2019), and (c) the items have high face validity (e.g., the item “The coursework is interesting to me” asks students about “interest”). The five factors explained 73.3% of the variance. The 0.0000106 value for the determinant of the correlation matrix was acceptable (Field, 2000), the 0.93 value for the Kaiser-Meyer-Olkin measure of sampling adequacy was “marvelous” (Kaiser, 1974), and the Bartlett test of sphericity was statistically significant (*χ*^2^[153] = 3,711.9, *p* < 0.001).

The Cronbach’s alpha values for all the measured variables were acceptable (George and Mallery, 2003), ranging from 0.79 to 0.93 (see Table 1). Descriptive statistics for each variable and correlations between the study variables are also provided in Table 1. The correlations among the MUSIC variables ranged from 0.48 to 0.73 and correlations between the MUSIC variables and behavioral and cognitive engagement, varied from 0.33 to 0.61.

**Table 1:** Correlations among Study 1 variables.

	1	2	3	4	5	6	7
1. Empowerment							
2. Usefulness	0.48						
3. Success	0.69	0.55					
4. Interest	0.68	0.60	0.73				
5. Caring	0.56	0.57	0.52	0.58			
6. Behavioral engagement	0.47	0.37	0.55	0.56	0.33		
7. Cognitive engagement	0.51	0.37	0.61	0.52	0.33	0.68	
*M*	4.71	5.00	4.38	4.47	5.31	4.19	3.70
*SD*	0.85	0.80	0.93	0.96	0.60	1.09	1.02
Cronbach’s *α*	0.79	0.79	0.87	0.89	0.82	0.92	0.93

*p* < 0.001 for all correlations.

**Figure 2:**
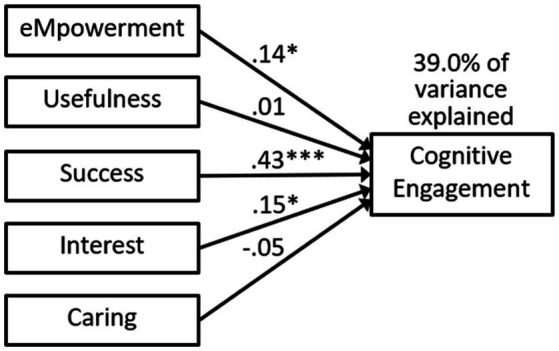
Model 1a regression predicting cognitive engagement. Statistics are standardized beta coefficients. * *p* < 0.05, *** *p* < 0.001.

**Figure 3:**
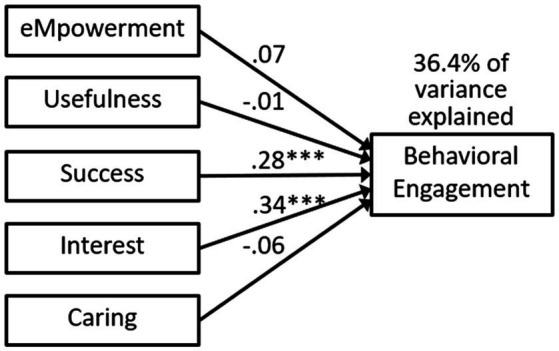
Model 1b regression predicting behavioral engagement. Statistics are standardized beta coefficients. *** *p* < 0.001.

We conducted regressions with students’ MUSIC perceptions predicting cognitive engagement in one model (Figure 2) and behavioral engagement in the other model (Figure 3). Success and interest were significant predictors of both cognitive and behavioral engagement. Empowerment was also a significant predictor of cognitive engagement. The MUSIC constructs explained 39.0% of the variance in cognitive engagement and 36.4% of the variance in behavioral engagement.

### Discussion

Our first two research questions led us to examine the extent to which students’ MUSIC perceptions were related to their cognitive engagement (RQ1) and behavioral engagement (RQ2). The correlations in Table 1 and significant paths in Figure 2 (Model 1a) and Figure 3 (Model 1b) indicate that students’ MUSIC perceptions were significantly related to both their cognitive and behavioral engagement. The reason we included all five of the MUSIC variables in one regression model for Models 1a and 1b was to determine whether some of the motivational climate variables were more important than others in predicting cognitive and behavioral engagement. Success, interest, and empowerment were statistically significant predictors of cognitive engagement, whereas success and interest were statistically significant predictors of behavioral engagement.

These findings provided evidence that students’ MUSIC perceptions are related to their cognitive and behavioral engagement in college English courses in China. Given these findings, we conducted a second study to determine whether these results were generalizable to students at a different university. Furthermore, we wanted to examine whether students’ cognitive and behavioral engagement predicted their achievement (RQ3, RQ4, and RQ5).

## Study 2

### Method

#### Participants

Participants in Study 2 were 259 undergraduates at a large university in mid-eastern China. The students were enrolled in one of six English classes (*n* was about 45 students per class) for non-English majors. More of the participants were female (*n* = 180; 69.5%) than male (*n* = 79; 30.5%). Most of the participants reported that they were Han nationality (*n* = 255, 98.5%), whereas four participants (1.5%) reported they were one of the minority nationalities. All the participants were 1st-year students who ranged in age from 18 to 21. Most or all students had been enrolled in English classes in school for at least 10 years (since elementary school).

#### Procedure

The procedures were similar to those in Study 1 except that students completed the questionnaire with paper-and-pencil (instead of online) near the end of the semester of their English course. Also, students gave their written consent to participate in the study prior to completing the questionnaire. These data were approved for inclusion in this study by the authors’ Institutional Review Board (IRB #16-932).

#### Measures

All the measures were the same as those used in Study 1. In addition, we used a measure of achievement that included students’ scores from the final English test that was administered at the end of their first college English course. This test was developed by instructors at another university to resemble (in structure and content) the national English test that students take after completing three courses in college English. Therefore, some of the content on the exam had not been covered in the course in which students were currently enrolled because this course was only their first college English course. The test included a writing section (students wrote an essay), a listening section (students listened to daily news and conversation in English and answered multiple-choice questions), a reading comprehension section (students read passages and then inserted paragraphs into the correct position in an article and answered multiple-choice and fill-in-the-blank questions), and a translation section (students translated a paragraph from Chinese to English). The range of possible test scores was 0–100.

#### Analysis

We used IBM® SPSS® version 26 to compute measures of dispersion, distribution, and correlation. IBM® SPSS® Amos™ version 25 was used to estimate the structural models with students’ MUSIC perceptions predicting cognitive engagement, behavioral engagement, and/or achievement as described in the “Results” section. We assessed the construct validity of the 18-item MUSIC Inventory used in Study 1 by conducting a confirmatory factor analysis (CFA). For all statistical tests, we set the alpha value at 0.05, and we report two-tailed values of *p*.

### Results

The results of the CFA for the 18-item MUSIC Inventory are presented here: *χ*^2^ = 289.82, *df* = 125, *p* < 0.001; and the fit indices indicated a reasonable fit to the data (Hu and Bentler, 1999; Kline, 2005), with CFI = 0.911, SRMR = 0.063, and RMSEA = 0.071 (90% confidence interval ranged from 0.061 to 0.082). In addition, the Cronbach’s alpha values for the MUSIC constructs, cognitive engagement, and behavioral engagement ranged from acceptable to excellent (see Table 2; George and Mallery, 2003). Descriptive statistics for each variable and correlations between the study variables are also provided in Table 2. The correlations among the MUSIC variables ranged from 0.22 to 0.59, which are slightly lower than the correlations obtained in Study 1. Similarly, the correlations between the MUSIC variables and cognitive and behavioral engagement were slightly lower than those in Study 1 and varied from 0.13 to 0.57. The scores on the achievement test ranged from 27 to 77 with a mean score of 55.9 (*SD* = 8.60). Achievement was significantly correlated with behavioral engagement, but not cognitive engagement (see Table 2).

**Table 2:** Correlations among Study 2 variables.

	1	2	3	4	5	6	7
1. Empowerment							
2. Usefulness	0.26						
3. Success	0.50	0.35					
4. Interest	0.49	0.39	0.59				
5. Caring	0.32	0.47	0.22	0.38			
6. Behavioral engagement	0.35	0.40	0.57	0.49	0.20		
7. Cognitive engagement	0.33	0.20	0.56	0.37	0.13^*^	0.60	
8. Achievement	0.09^NS^	0.13^*^	0.24	0.15^*^	0.09^NS^	0.18^*^	0.09^NS^
*M*	4.64	5.30	4.27	4.47	5.30	4.42	3.56
*SD*	0.67	0.71	0.82	0.73	0.52	0.80	0.84
Cronbach’s *α*	0.72	0.77	0.85	0.80	0.75	0.92	0.90

*p* < 0.001 for all correlations.

* Denotes *p < 0.05.*

NS Denotes *p* > 0.05 (not significant).

**Figure 4:**
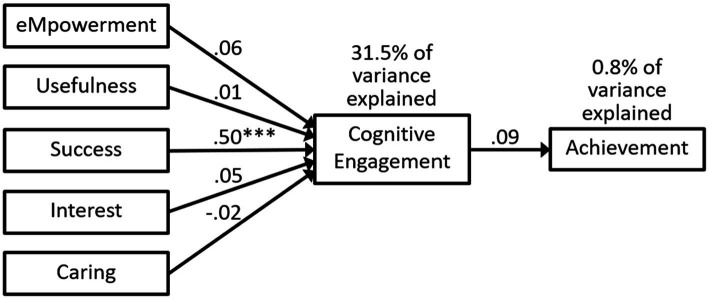
Model 2a: path analysis predicting cognitive engagement and achievement. Statistics are standardized beta coefficients. *** *p* < 0.001.

We analyzed the variables in the path analyses as observed constructs instead of latent constructs because the fit indices were not as good when we used latent constructs in the models. The fit indices were good for model 2a in Figure 4 that included the MUSIC constructs, cognitive engagement, and achievement (see Table 3). The MUSIC constructs explained 31.5% of the variance in cognitive engagement, whereas cognitive engagement only explained 0.8% of the variance in achievement (see Figure 4). Success was the only MUSIC construct significantly related to cognitive engagement in the path analysis (see Figure 4). The standardized indirect effects for the MUSIC constructs on achievement were also insignificant and the values were as follows: 0.006 for empowerment (*p* = 0.286), 0.001 for usefulness (*p* = 0.728), 0.044 for success (*p* = 0.161), 0.005 for interest (*p* = 0.332), and − 0.002 for caring (*p* = 0.466).

**Table 3:** Fit indices for the models in Figures 4–6.

Model	CFI	SRMR	RMSEA [90% CI]	*χ* ^2^
Model 2a	0.977	0.050	0.088 [0.039, 0.141]	15.01 (*df* = 5), *p* = 0.010
Model 2b	0.988	0.040	0.064 [0.000, 0.120]	10.29 (*df* = 5), *p* = 0.067
Model 2c	0.938	0.072	0.113 [0.081, 0.147]	47.20 (*df* = 11), *p* < 0.001

CFI, comparative fit index; SRMR, standardized root mean squared residual; RMSEA, root-mean-square error of approximation; and CI, confidence interval.

**Figure 5:**
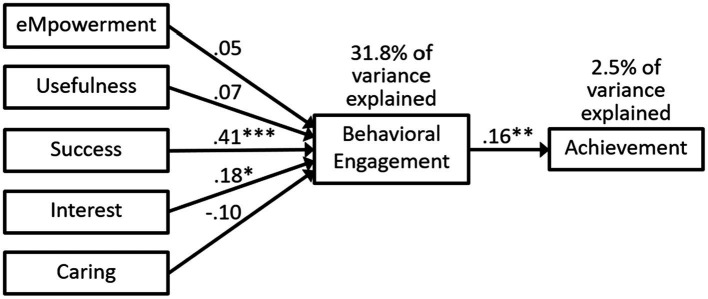
Model 2b: path analysis predicting behavioral engagement and achievement. Statistics are standardized beta coefficients. * *p* < 0.05, ** *p* ≤ 0.01, *** *p* < 0.001.

The fit indices were good for Model 2b in Figure 5 that included the MUSIC constructs, behavioral engagement, and achievement (see Table 3; Hu and Bentler, 1999; Byrne, 2001; Kline, 2005). The MUSIC constructs explained 31.8% of the variance in behavioral engagement, whereas behavioral engagement explained 2.5% of the variance in achievement (see Figure 5). Success and interest were the only two MUSIC constructs significantly related to behavioral engagement in the path analysis (see Figure 5). The standardized indirect effects for the MUSIC constructs on achievement were statistically significant for success (*p* < 0.01) and interest (*p* < 0.05), and borderline significant for caring (*p* = 0.05), with the following values: 0.008 for empowerment (*p* = 0.370), 0.010 for usefulness (*p* = 0.184), 0.064 for success (*p* = 0.006), 0.028 for interest (*p* = 0.016), and − 0.016 for caring (*p* = 0.050).

**Figure 6:**
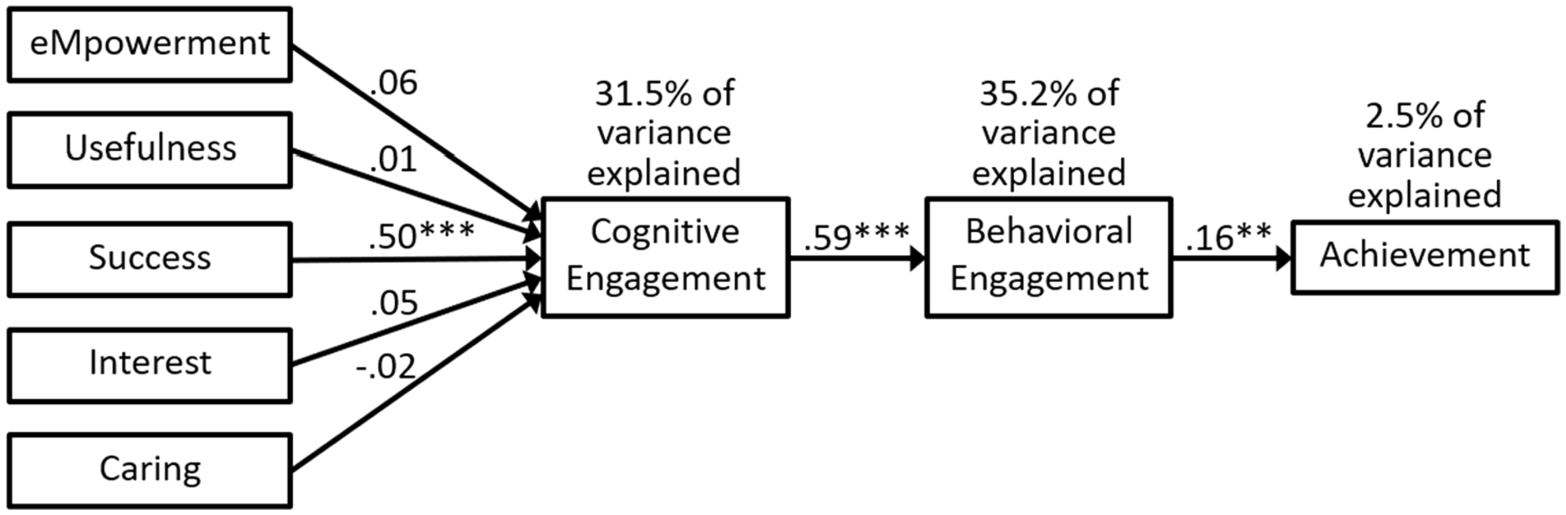
Model 2c: path analysis predicting cognitive and behavioral engagement, and achievement. Statistics are standardized beta coefficients. ** *p* ≤ 0.01, *** *p* < 0.001.

The fit indices for model 2c in Figure 6 that included the MUSIC constructs, cognitive and behavioral engagement, and achievement were reasonable for the CFI and SRMR values, but the RMSEA value was a little higher than acceptable (see Table 3). The MUSIC constructs explained 31.5% of the variance in cognitive engagement, cognitive engagement explained 35.2% of the variance in behavioral engagement, and behavioral engagement explained 2.5% of the variance in achievement. In the path analysis, success was the only MUSIC construct significantly related to cognitive engagement, cognitive engagement was significantly related to behavioral engagement, and behavioral engagement was significantly related to achievement (see Figure 6). The standardized indirect effects for the MUSIC constructs on behavioral engagement were statistically significant only for success (*p* < 0.001), with the following values: 0.038 for empowerment (*p* = 0.438), 0.004 for usefulness (*p* = 0.893), 0.259 for success (*p* < 0.001), 0.031 for interest (*p* = 0.546), and −0.041 for caring (*p* = 0.717). The standardized indirect effects for the MUSIC constructs on achievement were statistically significant only for success (*p* < 0.01), with the following values: 0.006 for empowerment (*p* = 0.312), 0.001 for usefulness (*p* = 0.829), 0.047 for success (*p* = 0.007), 0.005 for interest (*p* = 0.392), and − 0.002 for caring (*p* = 0.594).

### Discussion

#### Research Question 1 and 2

The purpose of this study was to investigate the relationships among students’ MUSIC perceptions, cognitive engagement, behavioral engagement, and achievement within the context of undergraduate English courses in China. Related to RQ1 (To what extent do students’ MUSIC perceptions relate to their cognitive engagement?) and RQ2 (To what extent do students’ MUSIC perceptions relate to their behavioral engagement?), the results from Study 2 are similar to those from Study 1 in that all five MUSIC perceptions were significantly correlated with cognitive and behavioral engagement. Furthermore, the MUSIC constructs explained a good amount of the variance in cognitive engagement (31.5%) and behavioral engagement (31.8%). In the path models, success was a significant predictor of cognitive engagement and success and interest were predictors of behavioral engagement. These findings are similar to Study 1 except that interest and empowerment did not predict cognitive engagement in Study 2 as they did in Study 1. In sum, we documented that students’ perceptions of the motivational climate (as measured by students’ MUSIC perceptions) were significantly related to cognitive and behavioral engagement. The success variable was the best predictor of cognitive engagement, and success and interest were the best predictors of behavioral engagement.

#### Research Question 3

Our third research question asked: To what extent do students’ MUSIC perceptions positively predict their cognitive engagement, which then positively predicts their achievement? To answer this question, we first examined the extent to which the data fit the structural model shown in Figure 4 (Model 2a). The data fit Model 2a reasonably well (see Table 3); however, the RMSEA value of 0.088 was a little high and above our pre-identified cutoff value of 0.08. In addition, the path from cognitive engagement to achievement was insignificant, and the indirect effects from all the MUSIC constructs to achievement were insignificant. Given these findings, the answer to RQ3 is that the success variable predicts cognitive engagement, but that cognitive engagement does not predict achievement; and therefore, Model 2a is not a good model.

#### Research Question 4

Our fourth research question asked: To what extent do students’ MUSIC perceptions positively predict their behavioral engagement, which then positively predicts their achievement? Figure 5 shows the model we tested to answer this question. The data fit Model 2b well (see Table 3) with all the fit indices meeting our criteria. Furthermore, the paths from success and interest to behavioral engagement were significant, as was the path from behavioral engagement to achievement. In addition, the indirect effects on achievement were significant for success (*p* < 0.01) and interest (*p* < 0.05) and borderline significant [*p* = 0.05] for caring. Thus, we conclude that Model 2b is a good model, which shows how students’ perceptions of success and interest significantly predict behavioral engagement, which then significantly predicts achievement.

#### Research Question 5

Our fifth research question included both cognitive and behavioral engagement in the same model and asked: To what extent do students’ MUSIC perceptions positively predict their cognitive engagement, which then positively predicts their behavioral engagement, which then positively predicts their achievement? Although the CFI and SRMR values were reasonable (see Table 3) for the model depicted in Figure 6 (Model 2C), the value for RMSEA (0.113) was higher than our preidentified cutoff value of 0.08; thus, making the overall fit not very good. As others have noted (e.g., Xia and Yang, 2019), using cutoff values for fit indices is not an exact science. Therefore, although the model fit was not great, it was pretty good. We then examined the significance of the paths between the constructs. The success construct was significantly related to cognitive engagement, which was significantly related to behavioral engagement, which was a significant predictor of achievement. Moreover, there was a significant (*p* < 0.001) indirect effect from success to achievement. Taken together, even though the fit for Model 2c was not as good as it could have been, we documented significant relationships among the constructs as predicted (although only success and not the other MUSIC perceptions were significantly related to cognitive engagement).

Although the path from behavioral engagement to achievement was significant, the magnitude of the standardized beta coefficient (*β* = 0.16) was relatively small. It is possible that the measure of achievement was insufficient to capture the *gains* in students’ abilities that occurred during the course because it was designed to measure students’ abilities at the end of three courses in college English. Because this was only the first course of a three-course sequence of courses, students had not learned all the skills needed to succeed on this test. As evidence, the mean score on the test was only slightly above 50% (*M* = 55.9; *SD* = 8.6) and the scores ranged from 27 to 77 out of a scale that ranged from 0 to 100. Nonetheless, the small standardized beta coefficient is similar in magnitude to those reported in other studies with undergraduates in the United States. For example, Jones et al. (2021) documented a standardized beta coefficient of 0.13 between students’ behavioral engagement and achievement. Jones and Carter (2019) reported a slightly higher standardized beta coefficient of 0.24 between behavioral engagement and learning; however, instead of using a measure of achievement, they used a measure of learning that controlled for students’ prior knowledge. Therefore, it was likely a better measure of what students learned in the course than the standardized achievement test used in the present study. In sum, compared to other studies, the magnitude of the relationship between behavioral engagement and achievement in the present study is fairly typical and is within the range of 0.00 to 0.30 that Reeve et al. (2020) noted as typical for studies linking engagement to educational outcomes.

## Limitations

The results of this study must be interpreted within the context of its limitations. Although the studies were conducted at two different universities, the results would be more generalizable if more universities and classes were included in the study. Another limitation was that instructors’ teaching strategies were not documented; therefore, we are not able to comment on how instructional practices may have influenced students’ perceptions. Some studies have made connections between instructional activities and students’ MUSIC perceptions (e.g., McGinley and Jones, 2014; Li et al., 2021; Jones et al., 2022a) and these types of analyses can be helpful to instructors who want to design instruction that motivates students. Finally, in the MUSIC model, external factors such as the culture can influence students’ MUSIC perceptions. However, the design of the present studies did not allow us to examine cultural influences on Chinese students’ MUSIC perceptions and engagement.

## Implications and Conclusions

The findings from these studies provide implications for researchers and practitioners interested in effective teaching approaches. We begin this section by discussing some of the theoretical implications for researchers who study the relationships between students’ motivational perceptions and engagement. Then, we discuss the implications as they relate to effective teaching.

## Theoretical Implications

Our findings add to the research studies that have included all the MUSIC constructs in one model to predict engagement. In our studies, success predicted cognitive engagement in both studies and empowerment and interest predicted cognitive engagement in Study 1. In another study with a sample of students very similar to the present studies (i.e., they associated students’ MUSIC perceptions with cognitive engagement in an EL class in China), Li et al. (2016) found that success and empowerment predicted cognitive engagement. In a different study with two groups of students, Li et al. (2021) documented that success, empowerment, and interest predicted cognitive engagement with one group of students, and usefulness, interest, and caring predicted cognitive engagement with another group of students who received a different type of instruction. Together, these findings indicate that while success, empowerment, and interest are generally associated with cognitive engagement, these associations can vary somewhat across different EL courses in China. Future studies could include more classes than the present study and be designed to determine whether systematic patterns of relationships between the MUSIC perception variables and engagement exist.

Theoretically, less is known about how the five MUSIC constructs are associated with each other within any one particular course. It is possible that some MUSIC constructs may be antecedents to others in which case increases in any one MUSIC construct could also lead to increases in one or more of the other MUSIC constructs. For example, in the self-determination theory (Ryan and Deci, 2020), constructs similar to empowerment (autonomy), success (competence), and caring (relatedness) are viewed as antecedents to intrinsic motivation, which is often defined similar to how interest was defined in the present study. Therefore, it is possible that empowerment and caring are important in supporting interest in our study. However, the relationships among the MUSIC perceptions are not always straightforward. Researchers studying interest have documented that increasing empowerment through choice can increase interest, but only when certain conditions are met. For example, in one study, choice increased interest, but only when individuals already had a high interest in the task and when the task was perceived as boring (Patall, 2013). Other studies have shown that empowering students through choice can enhance interest when initial success perceptions are high, but not to the same extent when success perceptions are lower (Patall et al., 2014). Perceptions of usefulness have also been found to be related to interest (Patall et al., 2013); yet perhaps only when students have low perceptions of success (Hulleman and Harackiewicz, 2009). Findings from studies such as these demonstrate the complex relationships that can occur between the MUSIC constructs. Although the present studies serve as a proof of concept to demonstrate that relationships exist among these constructs in English courses in China, further studies are needed to systematically examine whether any of the constructs serve as antecedents or whether there are interactions among the constructs.

## Teaching Implications

Because at least some of the MUSIC perceptions are related to engagement in all of these studies, EL instructors may be able to increase students’ engagement by implementing strategies that focus on these MUSIC perceptions. Based on the results of these studies, effective instructors could attempt to increase students’ perceptions of success, and also, possibly interest and empowerment. Although our findings are based on correlational analyses and do not imply causation, it is reasonable to suspect that EL instructors could increase students’ engagement by implementing success, interest, and empowerment strategies. Therefore, an implication is that effective EL instructors engage students by using strategies that increase their perceptions of success, trigger and maintain their interest, and empower them by giving them some choices within course activities and assignments.

For example, strategies that can lead to increases in students’ success expectancies include matching the difficulty levels of assignments with the abilities of the students; providing regular, specific feedback to students about their work on assignments; and clearly communicating expectations to students (Jones, 2018, p. 95). There are also many strategies that instructors can use to increase students’ situational interest in the course, such as creating activities that pique students’ curiosity, using novelty and variety, pacing lessons and lectures appropriately (not too slow or too fast), and limiting lecture time by incorporating more student-centered activities (Jones, 2018). Finally, instructors can empower students by providing them with choices during class and within assignments and incorporating learner-directed approaches (e.g., project-based learning, inquiry approaches). Combinations of these strategies have been shown to increase student engagement in EL courses in China. As an example, when Li et al. (2021) used a novel cell phone technology in class along with a student-centered class activity (students worked in groups to create a summary of what they were learning), students reported higher MUSIC perceptions and engagement than students in a control group that listened to a teacher’s lecture. More experimental studies (such as the study by Li et al., 2021) would be helpful to determine how specific instructional strategies can affect students’ MUSIC perceptions and engagement.

## Data Availability Statement

The raw data supporting the conclusions of this article will be made available by the authors, without undue reservation.

## Ethics Statement

The studies involving human participants were reviewed and approved by the Virginia Tech IRB. In Study 1, participants completed the survey as part of their normal class activities; and therefore, consent to participate in this study was not required. In Study 2, participants provided their written informed consent to participate in the study.

## Author Contributions

ML and YG are responsible for the data collection and the first draft. TW is responsible for the statistical analysis. BJ is responsible for the first, second, and third drafts. All authors contributed to the article and approved the submitted version.

## Funding

The authors would like to thank Shanghai University of Engineering Science and Virginia Tech’s Open Access Subvention Fund for supporting the open access publication of this article.

## Conflict of Interest

The authors would like to thank Shanghai University of Engineering Science and Virginia Tech’s Open Access Subvention Fund for providing the financial support needed for the open access publication of this article.

## Publisher’s Note

All claims expressed in this article are solely those of the authors and do not necessarily represent those of their affiliated organizations, or those of the publisher, the editors and the reviewers. Any product that may be evaluated in this article, or claim that may be made by its manufacturer, is not guaranteed or endorsed by the publisher.
